# Spatial Metabolomics Reveals Localized Impact of Influenza Virus Infection on the Lung Tissue Metabolome

**DOI:** 10.1128/msystems.00353-22

**Published:** 2022-06-22

**Authors:** Danya A. Dean, London Klechka, Ekram Hossain, Adwaita R. Parab, Krystin Eaton, Myron Hinsdale, Laura-Isobel McCall

**Affiliations:** a Department of Chemistry and Biochemistry, University of Oklahoma, Norman, Oklahoma, USA; b Laboratories of Molecular Anthropology and Microbiome Research, University of Oklahoma, Norman, Oklahoma, USA; c Department of Biology, University of Oklahoma, Norman, Oklahoma, USA; d Department of Microbiology and Plant Biology, University of Oklahoma, Norman, Oklahoma, USA; e Department of Physiological Sciences, Oklahoma State University, Stillwater, Oklahoma, USA; Princeton University

**Keywords:** IAV, chemical cartography, influenza virus, lung tissue, metabolomics, mouse, spatial metabolomics

## Abstract

The influenza virus (IAV) is a major cause of respiratory disease, with significant infection increases in pandemic years. Vaccines are a mainstay of IAV prevention but are complicated by IAV’s vast strain diversity and manufacturing and vaccine uptake limitations. While antivirals may be used for treatment of IAV, they are most effective in early stages of the infection, and several virus strains have become drug resistant. Therefore, there is a need for advances in IAV treatment, especially host-directed therapeutics. Given the spatial dynamics of IAV infection and the relationship between viral spatial distribution and disease severity, a spatial approach is necessary to expand our understanding of IAV pathogenesis. We used spatial metabolomics to address this issue. Spatial metabolomics combines liquid chromatography-tandem mass spectrometry of metabolites extracted from systematic organ sections, 3D models, and computational techniques to develop spatial models of metabolite location and their role in organ function and disease pathogenesis. In this project, we analyzed serum and systematically sectioned lung tissue samples from uninfected or infected mice. Spatial mapping of sites of metabolic perturbations revealed significantly lower metabolic perturbation in the trachea compared to other lung tissue sites. Using random forest machine learning, we identified metabolites that responded differently in each lung position based on infection, including specific amino acids, lipids and lipid-like molecules, and nucleosides. These results support the implementation of spatial metabolomics to understand metabolic changes upon respiratory virus infection.

**IMPORTANCE** The influenza virus is a major health concern. Over 1 billion people become infected annually despite the wide distribution of vaccines, and antiviral agents are insufficient to address current clinical needs. In this study, we used spatial metabolomics to understand changes in the lung and serum metabolome of mice infected with influenza A virus compared to uninfected controls. We determined metabolites altered by infection in specific lung tissue sites and distinguished metabolites perturbed by infection between lung tissue and serum samples. Our findings highlight the utility of a spatial approach to understanding the intersection between the lung metabolome, viral infection, and disease severity. Ultimately, this approach will expand our understanding of respiratory disease pathogenesis.

## INTRODUCTION

Influenza virus outbreaks are a continuous public health issue. Seasonal global epidemics caused by both influenza A viruses (IAV) and influenza B viruses cause 300,000 to 500,000 deaths each year (1). Vaccinations are the current method of prevention, but they fail to account for every possible viral strain. Antiviral drugs are used for treatment of IAV but are most effective within a short window during early infection. Additionally, some strains may have developed resistance to these drugs (2). While influenza A(H1N1)pdm09-infected intensive care unit patients treated with neuraminidase inhibitors have greater survival rates than untreated patients, one in four treated patients still die (3). These findings indicate a strong need for new treatments for IAV infection and the potential for host-targeted therapeutics to supplement antiviral agents. Their development, however, necessitates an understanding of disease pathogenesis, which remains incompletely elucidated for IAV.

We used metabolomics to identify and analyze metabolites affected by IAV infection. Metabolomics is a method of analysis that focuses on small molecules involved in biological processes. This technique allows us to gain insight into the host chemical response to viral infection. A comprehensive understanding of the relationship between host and virus could aid in the development of more effective prevention and treatment options. Previous studies applied metabolomic methods to lung tissue and serum during IAV infection. These studies found that nucleosides such as uridine, lipids such as sphingosine and sphinganine, and amino acid metabolites such as kynurenine are increased during infection in lung tissue (4). Additionally, carbohydrates such as mannitol, *myo*-inositol, and glyceric acid are decreased during active infection. However, the location of IAV within the respiratory tract is dynamic. Viral localization and location of tissue damage within the respiratory tract also influences disease symptoms, disease severity, and transmissibility of the infection (5–8). Thus, a spatial perspective is necessary with regard to IAV and tissue metabolism. Chemical cartography is an approach that combines liquid chromatography-mass spectrometry (LC-MS) with 3D visualizations, leading to detailed spatial maps of metabolite distribution compared to pathogen load, tissue damage, or immune responses (9–11). This approach, when applied to other infectious diseases, enabled the discovery of new treatments for these conditions (10). This method has been used to study the impact of cystic fibrosis on the local lung metabolome (12, 13) but had not previously been applied to IAV infection.

Using this method, we analyzed the distribution of small (*m/z* 100 to 1,500) metabolites within the infected trachea and lung in comparison to serum samples and to uninfected animals. We identified changes in the lung metabolome and determined limited overlap in metabolites perturbed by IAV infection between lung tissue and serum. Additionally, we identified several metabolites altered by infection, such as amino acids, lipids and lipid-like molecules, and nucleosides. Interestingly, some of these metabolites were differentially affected between lung positions, especially when comparing lung lobes to trachea.

## RESULTS

**FIG 1:**
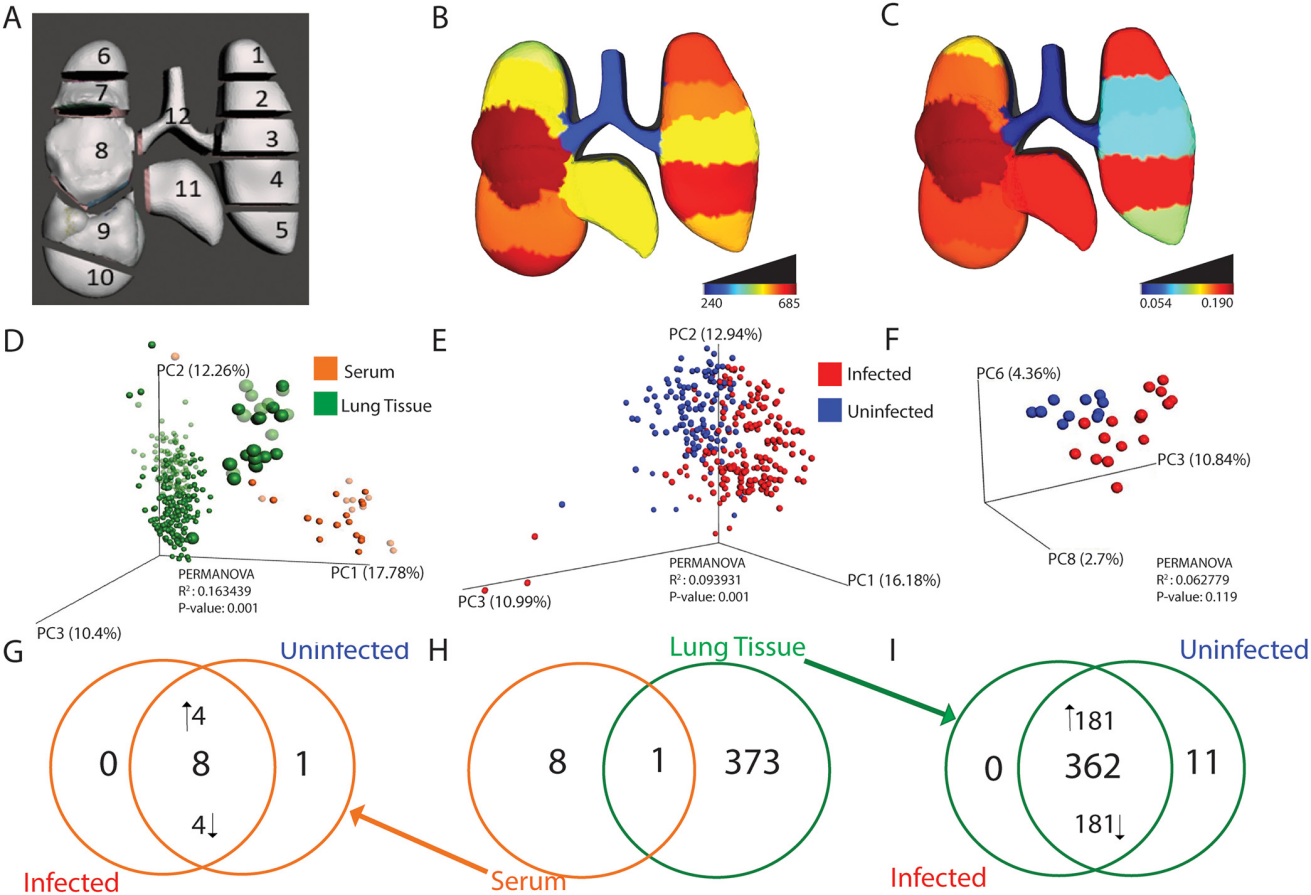
Localized impact of IAV infection in lung tissue and serum. *n* = 16 infected mice and *n* = 10 uninfected mice. (A) 3D model of lung tissue showing sampling positions. (B) Median viral burden distribution of IAV (expressed as relative luminescence units, RLU). Lower infection levels were observed in the trachea (Dunn’s test, FDR-corrected, *P* < 0.05 for comparisons between trachea and all positions except left lung middle [position 3]). (C) Magnitude of metabolic perturbation compared to uninfected (PERMANOVA R^2^). (D) Principal coordinate analysis (PCoA) plot showing differences in overall metabolome by sample type (PERMANOVA *P* value, <0.001). Green enlarged circles represent trachea samples. Faded circles represent uninfected samples. (E) Impact of infection on overall lung tissue metabolome (PERMANOVA *P* value, <0.001). (F) No significant impact of infection on the overall serum metabolome by PCoA. (G to I) Unique and common metabolites perturbed by infection. (G) Most metabolites perturbed by infection in serum samples are found in both infected and uninfected samples, albeit at different levels. Four of eight metabolites were increased by infection and four of eight were decreased by infection, while only one of the statistically significant metabolites was uniquely observed only in uninfected serum samples. (H) Overlap of metabolites perturbed by infection in serum (orange) or lung tissue all positions combined (green). (I) Most metabolites perturbed by infection in lung samples are found in both infected and uninfected samples, albeit at different levels (181/382 metabolites increased by infection and 181/362 decreased by infection, with 11 of the statistically significant metabolites uniquely detected in uninfected lung tissue). Panels C to I were generated from the full-feature table, generated, filtered, and processed as described in Materials and Methods, including both annotated and unannotated metabolite features.

Viral distribution influences IAV transmissibility, viral reassortment, and disease severity (5, 14, 15). While a few studies have investigated the changes in the metabolome during IAV infection, a spatial perspective of the metabolic disturbances is lacking (4, 16–18). We therefore used spatial metabolomics to identify candidate pathways and metabolites altered by infection in specific lung locations. Serum, trachea, and lungs were collected from IAV-infected mice at 3 days postinfection (*n* = 16 infected mice and *n* = 10 uninfected mice). Lungs were systematically sectioned into 11 segments based on lung physiology (Fig. 1A, Materials and Methods), and all samples were analyzed by liquid chromatography-tandem mass spectrometry (LC-MS/MS), followed by 3D reconstruction of metabolomics data. In addition, tissue homogenate bioluminescence was measured as an indicator of local viral burden in each tissue segment.

Using principal coordinate analysis (PCoA), we first compared lung and serum metabolomes and found that the local lung tissue metabolome does not reflect the circulating metabolome (permutational multivariate analysis of variance [PERMANOVA] *P* < 0.001, Fig. 1D). Furthermore, lung tissue overall was found to be greatly impacted by infection (PERMANOVA *P* < 0.001, R^2^ = 0.093931) (Fig. 1E). A similar but nonsignificant trend was seen between infected and uninfected serum samples (Fig. 1F).

Spatial analyses of lung tissue showed that viral load was largely localized to the lung tissue (positions 1 to 11) with minimal viral load in the trachea (position 12) (Dunn’s test *P* < 0.05, false-discovery rate [FDR]-corrected for comparisons between trachea and all positions except left lung middle [position 3, Fig. 1B]). The local magnitude of metabolic perturbation induced in response to infection was quantified using PERMANOVA R^2^ at each sampling site. PERMANOVA R^2^ indicates the percentage of the variance in the data can be explained by a specific metadata category (infection status here). The magnitude of metabolic perturbation was variable between tissue segments, with the highest R^2^ values in two segments of the left lung (position 1 and position 4) and the right lung middle lobe (position 8), whereas the trachea metabolome was least affected (R^2^ range from 0.05 in trachea to 0.18 at position 4; Fig. 1C; see Table S2 in the supplemental material). Thus, the site of lowest viral load matched with the site of lowest R^2^, i.e., the trachea. No significant differences were observed in viral load between the remaining tissue segments (Dunn’s test *P* > 0.05, FDR-corrected).

10.1128/msystems.00353-22.2 TABLE S2 R^2^ of metabolic perturbation in lung tissue. Download Table S2, XLSX file, 0.01 MB. Copyright © 2022 Dean et al. 2022 Dean et al. https://creativecommons.org/licenses/by/4.0/ This content is distributed under the terms of the Creative Commons Attribution 4.0 International license.

We next sought to determine whether the specific metabolites perturbed by infection differed between lung and serum (Fig. 1G to I). We used a random forest classifier, applied to serum on the one hand, and to lung tissue on the other hand (all positions combined). After applying significance cutoffs (see Materials and Methods), this approach yielded a total list of 9 serum metabolites and 374 lung tissue metabolites significantly perturbed by infection. There was strikingly limited overlap of infection-perturbed metabolites between lung tissue samples and serum samples, indicating that both sites respond differentially to infection (Fig. 1H) and concurring with overall PCoA analysis findings (Fig. 1D). We then sought to assess whether the metabolites perturbed by infection were uniquely elicited by infection or found under both conditions but at differential levels. The majority of infection-perturbed metabolites were common to infected and uninfected samples but found at different levels, with a minority uniquely detected in uninfected samples only (Fig. 1I). A similar trend was seen in infected and uninfected serum samples, where most infection-perturbed metabolites were present in both infected and uninfected samples, albeit at different levels (Fig. 1G). This signifies that the effect of IAV infection on the metabolome is primarily on metabolite levels rather than induction of novel metabolites.

**FIG 2:**
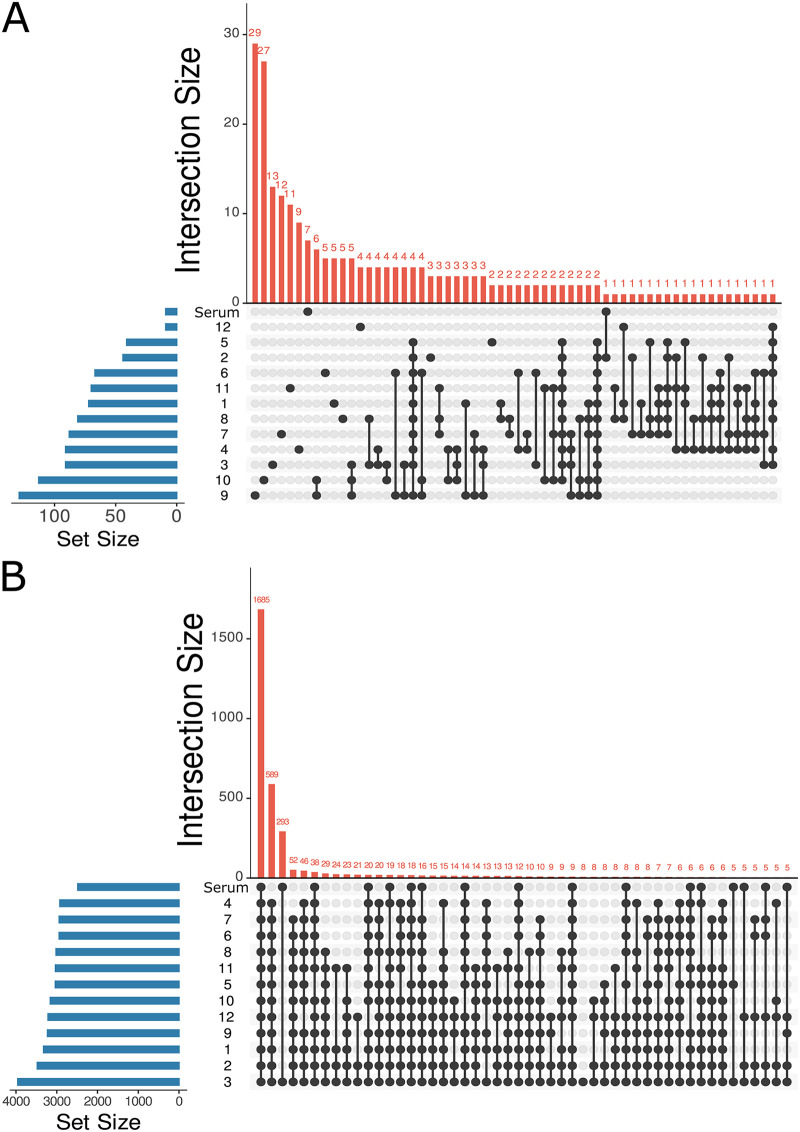
Location-specific impact of IAV infection on metabolism. *n* = 16 infected mice and *n* = 10 uninfected mice. Blue bars, number of metabolites present in each set; red bars, number of metabolites specific to each set or overlapping between sets; black circles, position at which metabolites are present; black circles connected via black line, overlapping metabolites or positions where metabolites intersect. (A) UpSet plot (number of intersections to show: 60) showing uniqueness of statistically significant infection-impacted metabolites in lung tissue and serum based on 13 random forest models analyzing each lung segment and serum separately. Order of sets (sampling sites) is based on set size, with no implications of similarity from set order. (B) UpSet plot (number of intersections to show: 50) of total metabolites found in lung tissue and serum showing large commonality across tissue sites and serum. Position numbers are as in Fig. 1A. The order of sets (sampling sites) is based on set size, with no implications of similarity from set order. Panels include both annotated and unannotated metabolite features.

Random forest classifier was also used to analyze the common and unique responses of each lung and tracheal tissue site to infection. A random forest model was built for each lung position and for serum, classifying infected versus uninfected samples. Strikingly, most infection-impacted metabolites were only affected at one or a few tissue sites rather than commonly across all or multiple lung lobe segments (Fig. 2A). This is not due to divergence in overall metabolome between sites, as analysis of all metabolites, irrespective of abundance, revealed large commonality across lung tissue sites and serum (Fig. 2B). This observation also indicates that the segregation between lung and serum in Fig. 1D is largely driven by differential metabolite abundance, rather than metabolite absence/presence. Likewise, infection-perturbed metabolites do not overlap appreciably with metabolites differing in abundance between lung and serum (Fig. S3). Very few perturbed metabolites were identified for position 12 (trachea), perhaps as a consequence of the low viral load at that site (Fig. 1B).

10.1128/msystems.00353-22.5 FIG S2 Representative mirror plots of metabolites perturbed by IAV infection in the lung tissue and serum. (A) Mirror plot of *m/z* 162.113, RT 0.297 min (top, black), to reference library spectrum (l-carnitine, bottom, green). (B) Mirror plot of *m/z* 147.076, RT 0.32 min (top, black), to reference library spectrum (glutamine, bottom, green). (C) Mirror plot of *m/z* 209.092, RT 0.631 min (top, black), to library spectrum (kynurenine, bottom, green). (D) Mirror plot of *m/z* 112.0508, RT 0.31 min (top, black), to reference library spectrum (cytosine, bottom, green). (E) Mirror plot of *m/z* 150.058, RT 0.323 min (top, black), to reference library spectrum (methionine, bottom, green). (F) Mirror plot of *m/z* 426.357, RT 2.982 min (top, black), to reference library spectrum (oleoyl l-carnitine, bottom, green). (G) Mirror plot of *m/z* 400.341, RT 2.952 min (top, black), to reference library spectrum (palmitoylcarnitine, bottom, green). (H) Mirror plot of *m/z* 616.175, 2.776 min (top, black), to reference library spectrum (hemin cation, bottom, green). (I) Mirror plot of *m/z* 198.085, RT 0.309 min (top, black), to reference library spectrum (l-citrulline, bottom, green). (J) Mirror plot of *m/z* 139.112, RT 2.644 min (top, black), to reference library spectrum (4-hydroxynonenal, bottom, green). (K) Mirror plot of *m/z* 146.165, RT 0.287 min (top, black), to reference library spectrum (spermidine, bottom, green). Download FIG S2, PDF file, 0.5 MB. Copyright © 2022 Dean et al. 2022 Dean et al. https://creativecommons.org/licenses/by/4.0/ This content is distributed under the terms of the Creative Commons Attribution 4.0 International license.

10.1128/msystems.00353-22.6 FIG S3 Limited overlap of metabolites differing in abundance between serum and lung tissue and metabolites impacted by infection in serum and at each lung position. Metabolites differing between serum and lung tissue were identified using random forest analysis, with a cutoff of >1 (“random forest” set). Likewise, metabolites differing between infected and uninfected samples were identified by random forest analysis at each tissue site and in serum individually, as in Fig. 2A (sets 1 to 12, representing lung segments 1 to 12, and serum). Download FIG S3, PDF file, 0.1 MB. Copyright © 2022 Dean et al. 2022 Dean et al. https://creativecommons.org/licenses/by/4.0/ This content is distributed under the terms of the Creative Commons Attribution 4.0 International license.

**FIG 3:**
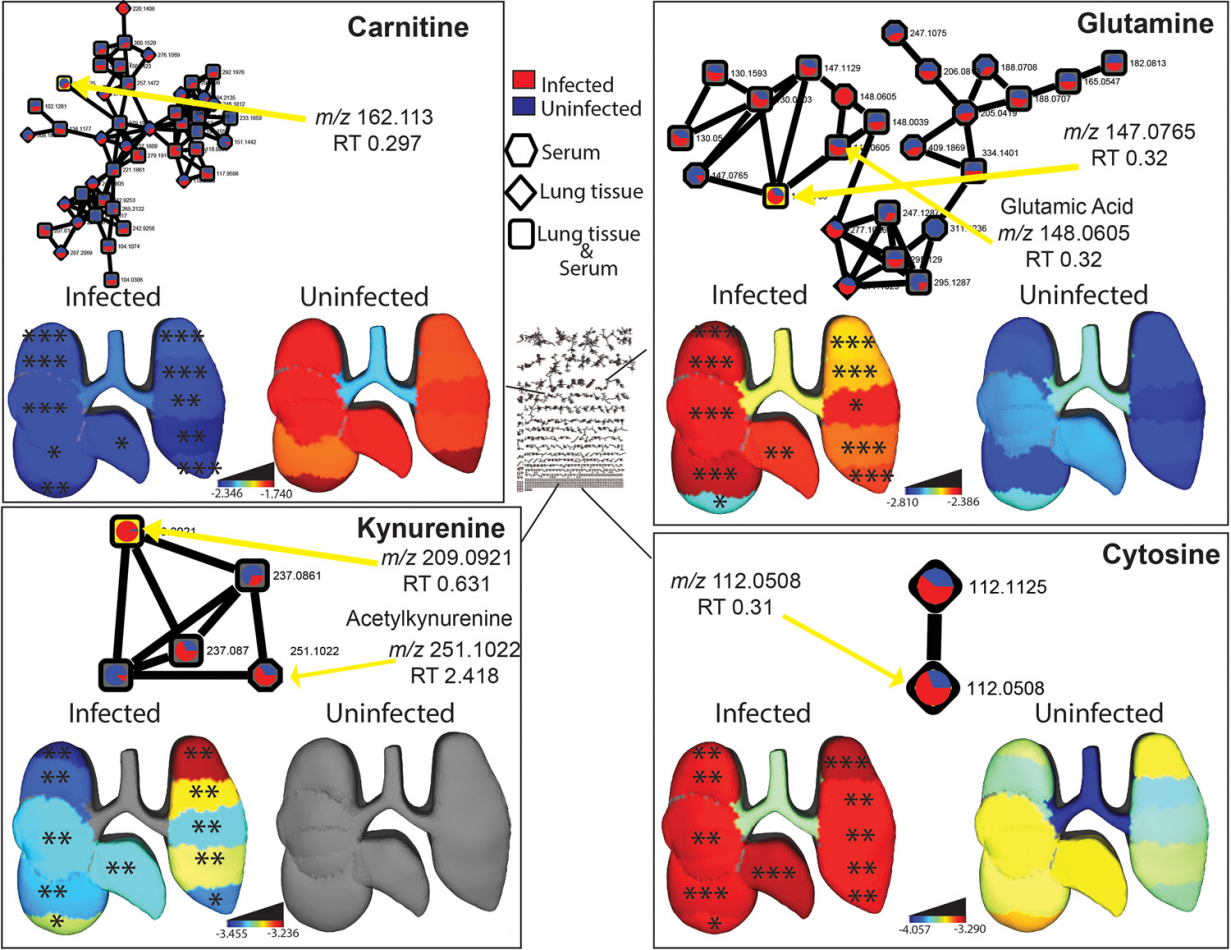
Representative metabolites perturbed by IAV infection. *n* = 16 infected mice and *n* = 10 uninfected mice. Molecular networks display the mean peak area of metabolites in infected (red) and uninfected (blue) samples. Each connected node represents structurally related metabolites, as determined by molecular networking. 3D lung ’ili plots show the log median of the TIC-normalized peak area of each displayed metabolite across tissue sites. Median values of zero remain uncolored (gray model background). Wilcoxon FDR-corrected *P* values comparing matched infected and uninfected lung tissue sites; *, *P* < 0.05; **, *P* < 0.01; ***, *P* < 0.001.

Metabolites perturbed by infection were annotated using molecular networking (19). Among these, carnitine, glutamine, kynurenine, and cytosine were found to be significantly and markedly perturbed by IAV infection and in a spatial manner (Fig. 3, Fig. S1). Glutamine, cytosine, and kynurenine were all significantly increased by IAV infection at all tissue sites except the trachea (Wilcoxon FDR-corrected *P* value, <0.05 at all sites except trachea) (Fig. 3, Fig. S1). The opposite trend was seen for carnitine, which was decreased by infection at all tissue sites except the trachea (Wilcoxon FDR-corrected *P* value, <0.05 at all sites except trachea) (Fig. 3, Fig. S1). In contrast, these metabolites were not significantly affected by infection in the serum. We further explored molecular networks for additional annotations. Specifically, the kynurenine (*m/z* 209.0921) neighbor *m/z* 251.1022 had no direct annotations but had a mass difference of 42.0101 to kynurenine, indicating acetylation (Fig. 3, bottom left panel). Thus, it was annotated as acetylkynurenine, though its differential abundance between infected and uninfected serum samples did not reach statistical significance (Fig. 3, bottom left). Acetylkynurenine could be a biological product of acetyltryptophan (20, 21), though we cannot exclude the possibility of it resulting from nonenzymatic breakdown of acetyltryptophan or from metabolite interconversion during extraction or LC-MS/MS data acquisition. In addition, the glutamine network neighbor glutamic acid (*m/z* 148.0605, retention time [RT] 0.32 min) was found significantly increased by infection (Wilcoxon FDR-corrected *P* value, <0.05 at all sites except position 10 and trachea) (Fig. 3, top right panel).

10.1128/msystems.00353-22.4 FIG S1 Boxplots of metabolites perturbed by IAV infection in lung tissue (red, infected; blue, uninfected). Carnitine statistically different at all positions except trachea (*P* value, <0.05). Glutamine statistically different at all positions except trachea (*P* value, <0.05). Kynurenine statistically different at all positions except trachea (*P* value, <0.05). Cytosine statistically different at all positions except trachea (*P* value, <0.05). Download FIG S1, PDF file, 0.6 MB. Copyright © 2022 Dean et al. 2022 Dean et al. https://creativecommons.org/licenses/by/4.0/ This content is distributed under the terms of the Creative Commons Attribution 4.0 International license.

**FIG 4:**
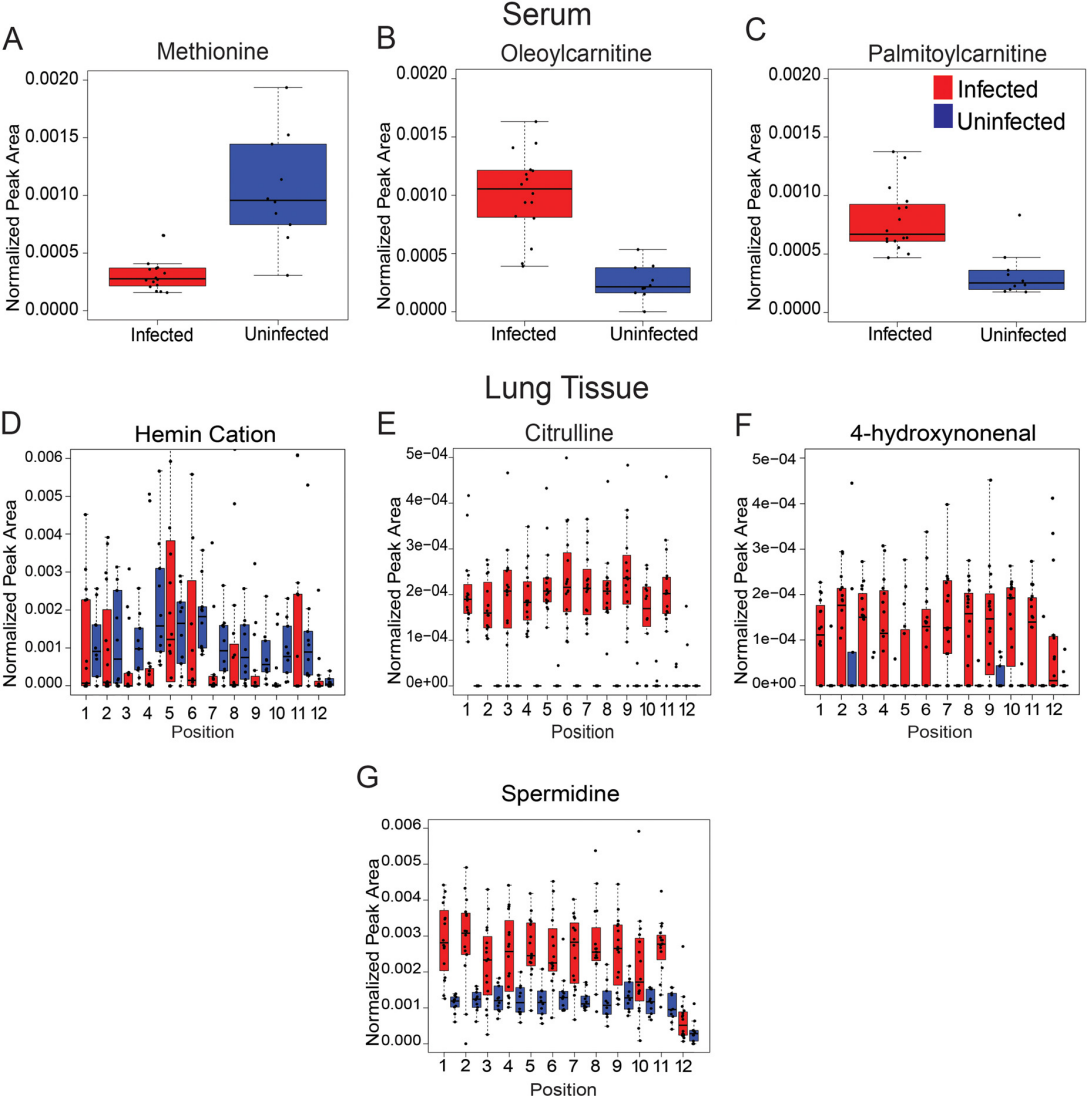
Representative metabolites perturbed by IAV infection in serum and lung tissue. *n* = 16 infected mice and *n* = 10 uninfected mice. (A to C) Statistically significant metabolites altered by infection in serum (Wilcoxon FDR-corrected *P* value, <0.001). (A) Methionine (*m/z* 150.058, RT 0.323 min). (B) Oleoylcarnitine (*m/z* 426.357, RT 2.982 min). (C) Palmitoylcarnitine (*m/z* 400.341, RT 2.952 min). (D to G) Statistically significant metabolites altered by infection in lung tissue. (D) Hemin cation (*m/z* 616.175 RT 2.776 min) statistically significant at positions 3, 4, 9, and 10 (Wilcoxon FDR-corrected *P* value, <0.05). (E) Citrulline (*m/z* 198.085, RT 0.309 min) statistically significant at all positions (Wilcoxon FDR-corrected *P* value, <0.05) except position 12. (F) 4-Hydroxynonenal (*m/z* 139.112, RT 2.644 min) statistically significant at all positions (Wilcoxon FDR-corrected *P* value, <0.05) except positions 2, 5, and 12. (G) Spermidine (*m/z* 146.165, RT 0.287 min) statistically significant at all positions (Wilcoxon FDR-corrected *P* value, <0.05) except positions 10 and 12.

Additional metabolites perturbed by infection in serum and lung tissue include amino acids, acylcarnitines, and nucleobases (Fig. 4, Table S1A and B), with contrasting effects between lung tissue positions and sample types. For example, amino acids had dissimilar effects following IAV infection in serum and lung tissue samples (Fig. 4): methionine was decreased in infected serum samples (Wilcoxon FDR-corrected *P* value, <0.001) while citrulline was increased in infected lung tissue samples (Wilcoxon FDR-corrected *P* value, <0.05) (Fig. 4A and E). Acylcarnitines (oleoylcarnitine and palmitoylcarnitine) were significantly increased in infected serum samples (Wilcoxon FDR-corrected *P* value, <0.001) compared to uninfected samples (Fig. 4B and C). Spermidine and 4-hydroxynonenal were significantly increased upon infection (Wilcoxon FDR-corrected *P* value, <0.05) at select lung tissue sites (Fig. 4F and G). Infection had the opposite effect on hemin, which was significantly decreased at positions 3, 4, 9, and 10 (Fig. 4D, Wilcoxon FDR-corrected *P* value, <0.05). In contrast, none of these metabolites were significantly affected by infection in the trachea.

10.1128/msystems.00353-22.1 TABLE S1 (A) Infection-perturbed metabolites in the lung (all positions combined). (B) Infection-perturbed metabolites in the serum. (C) Infection-perturbed metabolites in the lung at position 1. (D) Infection-perturbed metabolites in the lung at position 2. (E) Infection-perturbed metabolites in the lung at position 3. (F) Infection-perturbed metabolites in the lung at position 4. (G) Infection-perturbed metabolites in the lung at position 5. (H) Infection-perturbed metabolites in the lung at position 6. (I) Infection-perturbed metabolites in the lung at position 7. (J) Infection-perturbed metabolites in the lung at position 8. (K) Infection-perturbed metabolites in the lung at position 9. (L) Infection-perturbed metabolites in the lung at position 10. (M) Infection-perturbed metabolites in the lung at position 11. (N) Infection-perturbed metabolites in the lung at position 12. NA, not applicable. Download Table S1, XLSX file, 0.2 MB. Copyright © 2022 Dean et al. 2022 Dean et al. https://creativecommons.org/licenses/by/4.0/ This content is distributed under the terms of the Creative Commons Attribution 4.0 International license.

Given the differential viral load between the trachea and the remaining tissue sites, these metabolites may represent metabolic changes that require local viral load for induction. In contrast, several metabolites with no direct library matches (ClassyFire superclass-level annotation [22]: organic acids or derivatives, *m/z* 144.102, RT 0.311 min; *m/z* 247.202, RT 0.268 min; *m/z* 193.155, RT 0.314 min; *m/z* 261.217, RT 0.273 min; and *m/z* 158.118, RT 0.319 min (Table S1N) were similarly perturbed by infection in both the trachea and the lung lobes (Fig. 2A, Fig. S4E to I, Table S1N). This observation suggests a possible induction of these metabolic changes by infection status independent of local viral load. In contrast, four metabolites were uniquely perturbed by infection in the trachea (*m/z* 138.053, RT 0.299 min; *m/z* 166.084, RT 0.281 min; *m/z* 283.163, RT 0.275 min; and *m/z* 180.1, RT 0.278 min; Fig. 2A, Fig. S4A to D, Table S1N), which could indicate infection-adjacent metabolic changes that only occur at sites of low viral load (Fig. S4B) or that can only occur in the trachea and not in the lung lobes, likely representing trachea-specific or trachea-elevated metabolites (Fig. S4A, C, and D).

10.1128/msystems.00353-22.7 FIG S4 Metabolites perturbed by infection in trachea. *n* = 16 infected mice and *n* = 10 uninfected mice. (A to D) Statistically significant metabolites altered by infection in trachea (position 12) only (Wilcoxon FDR-corrected *P* value, <0.01). (E to I) Statistically significant metabolites altered by infection in both trachea (position 12) and lung lobes (positions 1 to 11). (E) Statistically significant at all positions (Wilcoxon FDR-corrected *P* value, <0.05). (F) Statistically different at all positions (Wilcoxon FDR-corrected *P* value, <0.05). (G) Statistically significant at all positions (Wilcoxon FDR-corrected *P* value, <0.05). (H) Statistically significant at all positions (Wilcoxon FDR-corrected *P* value, <0.05). (I) Statistically significant at all positions (Wilcoxon FDR-corrected *P* value, <0.05). Download FIG S4, PDF file, 1.2 MB. Copyright © 2022 Dean et al. 2022 Dean et al. https://creativecommons.org/licenses/by/4.0/ This content is distributed under the terms of the Creative Commons Attribution 4.0 International license.

## DISCUSSION

Here, we generated spatial maps of the metabolic impact of IAV infection on the mouse lung and trachea. We focused on female mice because female C57BL/6 mice have a more severe response to influenza infection than males (23) and due to the greater ease of cohousing female mice compared to males, which often have to be singly housed. Single housing increases stress on the animals, potentially confounding the results (24). Our approach revealed differential effects of infection across tissue sites and between lung and serum, as well as differential viral burden between trachea and lung tissue. Based on random forest analysis and molecular networking, infection-impacted metabolites of biological significance were annotated as acylcarnitines, amino acids, phospholipids, and nucleobases, among others.

Carnitine was significantly decreased by IAV infection at all lung positions except the trachea (Fig. 3, top left). Likewise, short-chain acylcarnitines such as CAR 2:0 and CAR 6:0 were also decreased in lung tissue (Table S1C to N). In contrast, in both serum and lung tissue, different long-chain acylcarnitines were increased by infection, including oleoylcarnitine (CAR 18:1) and palmitoylcarnitine (CAR 16:0) in serum and CAR 14:1 and CAR 20:1 in lung tissue (Fig. 4B and C, Table S1A to N). Carnitine and acylcarnitines are key intermediates in energy production via fatty acid beta oxidation (25). Influenza virus replication is sensitive to lipid metabolism inhibitors, supporting a causal role of these alterations in disease pathogenesis (26, 27). Likewise, these metabolic alterations may also be contributing to the differential responses to vaccination in obese versus lean animals (28). Oleoylcarnitine was also consistently elevated in serum from human H3N2 IAV infection compared to controls, from enrollment to 3 to 4 weeks postinfection (29).

Our study indicated that glutamine is increased upon IAV infection (Fig. 3, top right). These findings are consistent with studies in human nasal epithelial cells, which likewise observed strongly increased glutamine levels at 24 and 48 h postinfection (30). T cell proliferation and cytokine secretion rely heavily on the presence of glutamine (31). IAV-infected cells are also more dependent on glutamine availability than uninfected cells for survival (27), while glutamine treatment in the presence of bicarbonate inhibited IAV proliferation (32). The latter effect was observed only when treatment preceded viral addition, indicating an effect at early stages of the viral life cycle (32). In contrast, Thai et al. showed that inhibiting glutaminase inhibited IAV replication (33). The role of glutamine in IAV infection is thus complex and may differ depending on infection time point and cellular environment.

Citrulline was increased in infected lung tissue, in contrast with methionine, which was decreased in serum (Fig. 4A and D). Citrulline has downstream effects on T cell growth and response (34–36). Upregulation of citrulline suggests a role in overall immune response to IAV. Analyses of plasma from H1N1 pneumonia patients found an association with elevated methionine and mortality (37).

The nucleobase cytosine was elevated by infection in all lung sites except the trachea (Fig. 3, bottom right). Pyrimidine nucleotide biosynthesis is elevated upon IAV infection, and indeed, IAV replication was dependent on pyrimidine biosynthesis, with effects observed at early to intermediate stages of viral replication (38, 39). Inhibiting pyrimidine biosynthesis also overturned some of the effects of IAV on host mRNA export and inhibition of host antiviral gene expression (40). These findings indicate a proviral role of the observed cytosine elevation. Likewise, purine biosynthesis is also induced by IAV *in vitro* in human cells (41). Interestingly, cytosine was found to be discriminatory in the plasma between reverse transcriptase PCR (RT-PCR) COVID-positive patients and RT-PCR COVID-negative patients (42).

Several of the metabolites annotated and identified as infection-impacted in the lung tissue and serum in this study are congruent with prior studies of lung tissue during respiratory infection (4). Upregulation of cytosine in the lung tissue, oleoylcarnitine in serum, and several phospholipids in the lung tissue are consistent with current literature (Fig. 3, bottom right, Fig. 4B, Table S1A) (4, 17, 43). Amino acids and nucleosides, nucleotides, and analogs, in particular, were upregulated in lung tissue in other IAV studies as well as in Mycobacterium tuberculosis infection (TB) and respiratory syncytial virus (RSV) studies (4, 43–45). Citrulline is elevated in mouse lung tissue infected with RSV and in mice infected with TB (4, 17, 44–46). This coincides with our findings showing elevations of this amino acid in IAV-infected lung tissue (Fig. 4E). Glutamine was elevated in mouse lung tissue infected with TB, corresponding with our study (Fig. 3, top right) (45, 46).

Kynurenine, an infection-induced anti-inflammatory molecule, is consistently upregulated in mouse lung tissue of several respiratory infection studies, including TB, RSV, and our IAV study (Fig. 3, bottom left) (4, 43, 44, 46), as well as at 24 and 48 h postinfection in human nasal epithelial cells (30). Using different anti-IAV compounds that target different stages of the viral life cycle demonstrated that viral entry is the key stage responsible for induction of kynurenine in human macrophages (47). This may account for the fact that we did not observe elevated kynurenine in the trachea, where viral load was low. However, a disconnect in the lung between viral load and the activity of host indoleamine 2,3-dioxygenase (IDO), the enzyme responsible for kynurenine biosynthesis, indicates that elevation of kynurenine reflects host-mediated mechanisms, rather than direct induction by the virus (48). Indeed, IDO is induced in response to interferon signaling (49). A causal role for kynurenine in disease progression is further supported by findings of a higher kynurenine to tryptophan ratio in influenza patients with longer symptom duration or in patients who died of influenza infection compared to those who recovered (50). However, kynurenine lacks direct anti- or proviral effects, since inhibition of kynurenine production did not affect lung viral load in mouse models. Instead, inhibition of kynurenine production expedited tissue repair (51).

Analysis of circulating metabolites has been performed in multiple studies on IAV vaccinology (52, 53). Our findings of differential impacts of infection on lung and serum metabolites (Fig. 1H, Table S1A and B) indicate that the metabolic changes observed in those studies may not be directly linked to lung metabolic patterns, and this discrepancy was not due purely to differences between serum and lung overall (Fig. 2B). Likewise, the majority of COVID-19 metabolomic studies have relied on serum or plasma samples (54). By extension, based on this study’s results, they may not be relevant to the metabolic changes occurring in the lung, hampering translatability for drug development purposes.

We also observed differential impacts of infection across lung sections (Fig. 2A). The trachea, in particular, was especially divergent from the lung lobes in terms of viral burden (Fig. 1B), overall magnitude of infection-induced metabolic perturbations (Fig. 1C), and specific metabolic changes (Fig. 3 and 4, Fig. S4). Several factors likely contribute to this differential response to infection. Physiological differences in baseline tissue physiology are likely involved, given the fact that the trachea is divergent from the lung lobes in uninfected samples (Fig. 1D). Metabolic alterations in response to infection in the trachea only may either be reflective of this differential physiology or reflect changes in response to signals produced by infected cells, but that are dampened when the virus is present (perhaps as a means to dampen antiviral responses). Strikingly, some metabolites were commonly perturbed across the lung lobes and trachea, indicating that they are likely induced by host processes, rather than directly by local viral infection (Fig. S4). In contrast, metabolites perturbed by infection only in the lung lobes and not the trachea may reflect a need for local viral replication for induction of these metabolic changes. Further work will be necessary to confirm experimentally whether these locally modulated metabolites causally contribute to IAV infection progression and/or disease severity.

As with any untargeted metabolomics studies, a significant fraction of infection-impacted metabolites could not be annotated (~65%). The most commonly observed metabolite subclasses in our data set overall were amino acids, peptides and analogues, glycerophosphocholines, amines, and fatty amides. Although this represents a broad diversity of metabolite classes, nevertheless, complementary metabolite extraction or data acquisition methods could further expand this list.

We further acknowledge that the mouse model of influenza virus infection and mouse-adapted influenza viral strain may not be the most representative of the functional lung alterations that would occur during human infection (55). Indeed, the predominance of lower respiratory tract metabolic alterations over changes in the trachea observed in this study may be a consequence of this model. While accessing human lung samples for metabolomics is challenging, several metabolic alterations observed in human serum or during *in vitro* infection of human cells concur with our findings, as described above (29, 30, 50), thus supporting our results.

A further limitation is that we focused on a single time point. However, recent time course analysis data by van Liempd et al. demonstrated that many metabolic pathways altered in the serum at 3 days postinfection remain perturbed at 30 days postinfection, after the virus has been cleared and animal weight has recovered. Of relevance for this study, van Liempd et al. reported comparable acylcarnitine and carnitine metabolic alterations at both time points. In contrast, both our analyses and van Liempd et al. observed depletion of serum indolepropionic acid at the 3 days postinfection time point, which was followed in their analyses by restoration of indolepropionic acid levels back to baseline at 30 days postinfection (dpi) (56). These findings concur with observations of persistently perturbed metabolism in humans even up to 4 weeks after infection (29). Jointly, these reports indicate that these metabolic perturbations mainly result from host mechanisms rather than direct induction by the virus.

We anticipate our findings to serve as a resource upon which the research community can build to study the impact of different disease modifiers on the relationship between spatial changes in the lung metabolome and disease severity, for example, the impact of age, comorbidities, or treatment. Our findings and our approach also serve as a framework to study how the metabolome is restored in a spatially dependent fashion during recovery from respiratory infection, or fails to recover during chronic disease in additional disease models, and to identify markers of treatment response and infection outcome. Overall, we anticipate our approach to be broadly applicable to many other respiratory infections, helping expand our understanding of respiratory disease pathogenesis.

## MATERIALS AND METHODS

### *In vivo* infection.

All vertebrate animal studies were performed under a protocol approved by the Oklahoma State University Institutional Animal Care and Use Committee (protocol number VM20-36), in accordance with the USDA Animal Welfare Act and the Guide for the Care and Use of Laboratory Animals of the National Institutes of Health. Mice were housed throughout the experiment in an AAALAC-accredited vivarium (Association for Assessment and Accreditation of Laboratory Animal Care accreditation) under specific-pathogen-free conditions with *ad libitum* access to water and normal mouse chow. Caging was HEPA-filter ventilated.

Female C57BL/6J mice (Jackson Laboratories) were anesthetized with ketamine and xylazine intraperitoneally and then intranasally inoculated at 12 weeks of age with PR8-Glu (57) (a generous gift from Peter Palese, Icahn School of Medicine at Mount Sinai, Department of Microbiology, New York, NY, USA) at 2 × 10^3^ PFU/mouse in 25 to 50 μL phosphate-buffered saline (PBS). Controls were inoculated with PBS alone. Body weights were monitored daily (Fig. S5). Then, 3 days later, mice were anesthetized with ketamine and xylazine and injected intravenously (i.v.) with a working solution of coelenterazine (GoldBio, St. Louis, MO) made according to a standard protocol (58). Briefly, using a stock solution of 7.5 mg/mL in acidified alcohol, each mouse was administered 98 μg substrate in PBS (13 μL stock in 137 μL PBS) into the retroorbital sinus. After substrate administration, the thoracic cavity was opened followed by exsanguination by cardiac blood collection for serum and by lung tissue harvesting. Section sizes were determined based on mouse anatomy and to generate consistent tissue segments 10 to 50 mg in weight (Fig. 1A). We therefore sectioned the right lung by lobes (superior lobe, middle lobe, inferior lobe, postcaval lobe). The two larger right lung lobes (superior and inferior) were subsequently divided into upper and lower segments. The left lung was sectioned into five segments from the upper region to the lower region, with the central segment corresponding to the connection with the left bronchus. The upper trachea, cranial to tracheal bifurcation, was also collected. Subsequently, samples were submerged in coelenterazine (0.3 mg/mL) in a 96-well plate. Once collected, the tissue pieces were blotted and flash-frozen in liquid nitrogen for later analyses.

10.1128/msystems.00353-22.8 FIG S5 Mouse body weight over experiment duration. Day 2 weight data are missing for mice 18, 19 (infected), and 20 (uninfected) due to weather-related campus closure. Day 0, day 1, and day 3 data represent all animals in the study. Download FIG S5, PDF file, 0.01 MB. Copyright © 2022 Dean et al. 2022 Dean et al. https://creativecommons.org/licenses/by/4.0/ This content is distributed under the terms of the Creative Commons Attribution 4.0 International license.

Samples represent 6 independent cohorts of mouse infection. Each cohort included 2 to 3 infected mice and 1 to 3 uninfected mice, except for cohort 3, which only included two infected mice, for a total of *n* = 16 infected mice and *n* = 10 uninfected mice across all cohorts. The breakdown into multiple cohorts was necessary due to the detailed tissue sectioning, to ensure that endpoints were at comparable time points postinfection between mice.

### Metabolite extraction and UHPLC-MS/MS.

Metabolite extraction was performed jointly for all tissue samples, once samples across all cohorts had been collected. A two-step metabolite extraction procedure was performed according to Want et al. (59) for both tissue and serum samples. Samples were homogenized in LC-MS-grade water with steel beads utilizing a Qiagen TissueLyser at 25 Hz for 3 min, and 1 μL was removed for luminescence analysis, with the water volume normalized based on tissue weight at a constant ratio of 500 μL water/50 mg sample. Methanol spiked with 4 μM sulfachloropyridazine internal standard was added for a final concentration of 50%, and samples were homogenized again for 3 min and centrifuged at 16,000 × *g* for 10 min at 4°C. The supernatant (aqueous extract) was collected, dried overnight in a SpeedVac device, and frozen at −80°C until LC-MS analysis. The pellet produced from centrifugation was collected for organic extraction via addition of 3:1 dichloromethane:methanol spiked with 2 μM sulfachloropyridazine internal standard, at a standard ratio of 1,000 μL volume of solvent per 50 mg tissue weight, homogenized for 5 min, and centrifuged for 10 min at 4°C. The organic extract was air-dried overnight and then frozen at −80°C until LC-MS analysis. Dried aqueous and organic extracts were resuspended in 1:1 methanol:water spiked with the internal standard sulfadimethoxine and combined. Samples were then sonicated and centrifuged, and the supernatant was collected for analysis. A Thermo Scientific Vanquish ultra-high-performance liquid chromatography (UHPLC) system was used for tissue and serum analysis using a Kinetex 1.7-μm C8 100-Å LC column (50 × 2.1 mm). Chromatography was done with water plus 0.1% formic acid (mobile phase A) and acetonitrile plus 0.1% formic acid (mobile phase B), at a 0.5 mL/min flow rate (7.5 min) with a 40°C column temperature. The LC gradient can be found in Table 1. Data acquisition was performed in random sample order, with a blank and pooled quality control every 12 samples. To monitor instrumental drift, a 6-mix solution with 6 known molecules was run at the beginning and end of the LC/MS/MS analysis. Calibration of the instrument was also done immediately prior to instrument analysis using Pierce LTQ Velos ESI positive ion calibration solution. MS/MS detection was conducted on a Q Exactive Plus (Thermo Scientific) high-resolution mass spectrometer (Table 2). Ions were generated for MS/MS analysis in positive mode.

**TABLE 1:** LC gradient

Time (min)	Flow (mL/min)	% mobile phase B	Curve
0.000	Run		
0.000	0.500	2.0	5
1.000	0.500	2.0	5
2.500	0.500	98.0	5
4.500	0.500	98.0	5
5.500	0.500	2.0	5
7.500	0.500	2.0	5
7.500	Stop run		

**TABLE 2:** Q Exactive Plus (Thermo Scientific) instrument parameters

Parameter	Value
Runtime	0 to 7.5 min
Polarity	Positive
Default charge state	1
Full MS	
Resolution	70,000
AGC target^*a*^	3e6
Maximum IT	246 ms
Scan range	100 to 1500 *m/z*
dd-MS2/dd-SIM	
Resolution	17,500
AGC target	1e5
Maximum IT	54 ms
Loop count	5
TopN	5
Isolation window	1.0 *m/z*
(N)CE/stepped (N)CE	NCE: 20, 40, 60
dd Settings	
Minimum AGC target	8.00e3
Intensity threshold	1.5e5
Peptide match	Preferred
Exclude isotopes	On
Dynamic exclusion	10.0s
Tune data	
Spray voltage (+)	3800.00
Capillary temp (+ or +–)	320.00
Sheath gas (+ or +–)	35.00
Aux gas (+ or +–)	10.00
Spare gas (+ or +–)	0.00
Max spray current (+)	100.00
Probe heater temp (+ or +–)	0.00
S-lens RF level	50.00
Ion source	HESI

a AGC, Automatic Gain Control; IT, injection time; dd-MS2/dd-SIM, data-dependent MS2/data-dependent selected ion monitoring; NCE, normalized collision energy; RF, radiofrequency.

### Luminescence analysis.

Coelenterazine was prepared according to GoldBio standard protocols. Briefly, 1 mg coelenterazine was added to 1 mL of acidified methanol to make a stock solution. The stock solution was made to a final concentration of 1.5 μM. A 1:10 solution of sample homogenate to colentrazine was analyzed on a GloMax Explorer (Promega).

### LC-MS data analysis.

Data analysis was performed using MZmine version 2.53 (60), according to the parameters in Table 3, to develop the feature table for analysis. Blank removal with a 3-fold threshold was performed, and Jupyter Notebook was used to perform total ion current (TIC) normalization. Principal coordinate analysis (PCoA) was performed on the TIC-normalized MS1 feature table (Table S3) using the Bray-Curtis dissimilarity metric in QIIME version 2 (61). 3D PCoA plots were visualized using EMPeror (62). The lung 3D model was developed using Sketchup and MeshLab, and visualization was completed using ’ili (http://ili.embl.de/) (63).

**TABLE 3:** MZmine 2.53 parameters

Parameter	Value
MS^1^	
Retention time (min)	0–7.5
Noise level	5E5
MS²	
Retention time (min)	0–7.5
Noise level	1E3
Chromatogram builder	
Mass list	Masses
Minimum time span (min)	0.01
Minimum height	1.5E6
*m/z* Tolerance (ppm)	10.0
Chromatogram deconvolution	
Algorithm	Baseline cutoff
*m/z* Range for MS² scan pairing (Da)	0.001
RT range for MS² Scan pairing (min)	0.2
Minimum peak height	1.5E6
Peak duration range (min)	0–3.5
Baseline level	5E5
Deisotoping	
*m/z* tolerance (ppm)	10.0
Retention time tolerance (min)	0.5
Monotonic shape	Yes
Maximum charge	3
Representative isotope	Most intense
Alignment	
*m/z* Tolerance (ppm)	10.0
Weight for *m/z*	1
Weight for retention time	1
Retention time tolerance (min)	0.5
Row filtering	
Minimum peaks in a row	3
Retention time (min)	0.2–6.5
Keep only peaks with MS² scans	Yes
Reset peak no. ID	Yes

10.1128/msystems.00353-22.3 TABLE S3 Metabolite feature table (TIC-normalized peak areas). Download Table S3, CSV file, 5.9 MB. Copyright © 2022 Dean et al. 2022 Dean et al. https://creativecommons.org/licenses/by/4.0/ This content is distributed under the terms of the Creative Commons Attribution 4.0 International license.

Random forest analysis was conducted using R in Jupyter Notebook. The number of trees was restricted to 500, and the random forest classifier cutoff was based on a variable importance score of mean decrease accuracy of >1. Lists were further restricted to an FDR-corrected Mann-Whitney *P* value less than 0.05 and fold change of <0.05 or >2.0. Venn diagrams and UpSet plots were developed to quantify metabolite overlap within lung tissue positions and between lung tissue and serum using Intervene Shiny App (64).

Global Natural Products Social Molecular Networking (GNPS) (19) was used to perform feature-based molecular networking (19, 65), and MolNetEnhancer (66) was used according to the parameters in Table 4. MolNetEnhancer assigns a consensus ClassyFire (22) ontology to each molecular networking subnetwork based on annotated nodes, thus enabling the extension of ClassyFire ontology to nodes without direct annotations (66). Cytoscape 3.8.2. was used to visualize all molecular networks (67). All reported annotations are at Metabolomics Standards Initiative confidence level 2 (specific metabolite name provided) or level 3 (metabolite family name provided only) (68). Lipids were annotated based on the GNPS library or analog matches and using standard LIPID MAPS nomenclature (69).

**TABLE 4:** GNPS parameters

Feature-based molecular network parameter	Value
Precursor ion mass tolerance	0.02 Da
Fragment ion mass tolerance	0.02 Da
Minimum matched fragment ions	4
Maximum connected component size (beta)	100
Maximum shift between precursors	500 Da
Library search min matched peaks	4
Search analogs	Do search
Top results to report per query	1
Score threshold	0.7
Maximum analog difference	100.0 Da
Minimum peak intensity	0.0
Filter precursor window	Filter
Filter peaks in 50 Da window	Filter
Filter library	Filter
Normalization per file	Row sum normalization (per file sum to 1,000,000)
Aggregation method for peak abundances per group	Mean
PCoA	Bray-Curtis
Run dereplicator	Run

### Data availability.

All metabolomics data are publicly available in MassIVE under accession number MSV000085389 (ftp://massive.ucsd.edu/MSV000085389). The MolNet Enhancer link is https://gnps.ucsd.edu/ProteoSAFe/status.jsp?task=f9f194c9b723409f8927c84470f9a0c5, and the original GNPS link is https://gnps.ucsd.edu/ProteoSAFe/status.jsp?task=bb7a5f7fb32045208b6040908a1453f7.

10.1128/msystems.00353-22.9 FIG S6 Method validation. (A) Proportional relationship between injection volume and peak area for representative features. Pooled samples (pooled quality control, pooled QC) were analyzed by LC-MS as described for samples in Materials and Methods, but with injection volumes serially ranging from 5 μL to 30 μL. Data were processed in MZmine (60) as described for samples in Materials and Methods, and the relationship between injection volume and peak area for select metabolite features of interest was plotted in Excel. A proportional, linear relationship was observed between injection volume and peak area in all cases. The inset in each plot displays the Excel linear trendline R^2^. (B to D) Limited impact of drying time on representative metabolite features. C2C12 cells (three independent cultures) were extracted using the same method as for lung tissue samples. Each extract was split and either dried for only 3 h or overnight, following which, dried extracts were immediately stored at −80°C until LC-MS analysis. The resulting data were processed in MZmine (60) as described in Materials and Methods, features shared with blanks were removed, and data were TIC-normalized. Normalized peak areas of representative metabolite features are displayed. All comparisons between drying times are *P* > 0.05 by Mann-Whitney test (nonsignificant). (E) Comparable peak area for sulfadimethoxine (*m/z* 311.080, RT 2.52 min) internal standard in blanks and pooled quality control samples (pooled QC), same injection volume. *P* = 0.7017 by Mann-Whitney U test. Download FIG S6, PDF file, 1.2 MB. Copyright © 2022 Dean et al. 2022 Dean et al. https://creativecommons.org/licenses/by/4.0/ This content is distributed under the terms of the Creative Commons Attribution 4.0 International license.

10.1128/msystems.00353-22.10 FIG S7 Extracted ion chromatograms comparing known metabolite standards to annotated sample metabolites. Black, standard; red; sample. (A) Kynurenine (*m/z* 209.092, RT 0.631 min). (B) Adenosine-5-diphosphate (*m/z* 428.037, RT 0.441 min). (C) Aspartate (*m/z* 134.045, RT 0.322 min). (D) Citrulline (*m/z* 198.085, RT 0.309 min). (E) Cytidine (*m/z* 244.093, RT 0.307 min). (F) Cytosine (*m/z* 112.0508, RT 0.31 min). (G) Glutamine (*m/z* 147.076, RT 0.32), (H) Carnitine (*m/z* 162.113, RT 0.297). (I) Methionine (*m/z* 150.058, RT 0.323). (J) CAR2:0 acetylcarnitine (*m/z* 204.123, RT 0.316 min). (K) Palmitoylcarnitine (*m/z* 400.341, RT 2.952). Download FIG S7, PDF file, 0.6 MB. Copyright © 2022 Dean et al. 2022 Dean et al. https://creativecommons.org/licenses/by/4.0/ This content is distributed under the terms of the Creative Commons Attribution 4.0 International license.
