# Supplementary figures and images for: Spatial Metabolomics Reveals Localized Impact of Influenza Virus Infection on the Lung Tissue Metabolome
Source: mSystems. 2022 Jun 22;7(4):e00353-22. doi: 10.1128/msystems.00353-22 (PMC9426520; doi:10.1128/msystems.00353-22)

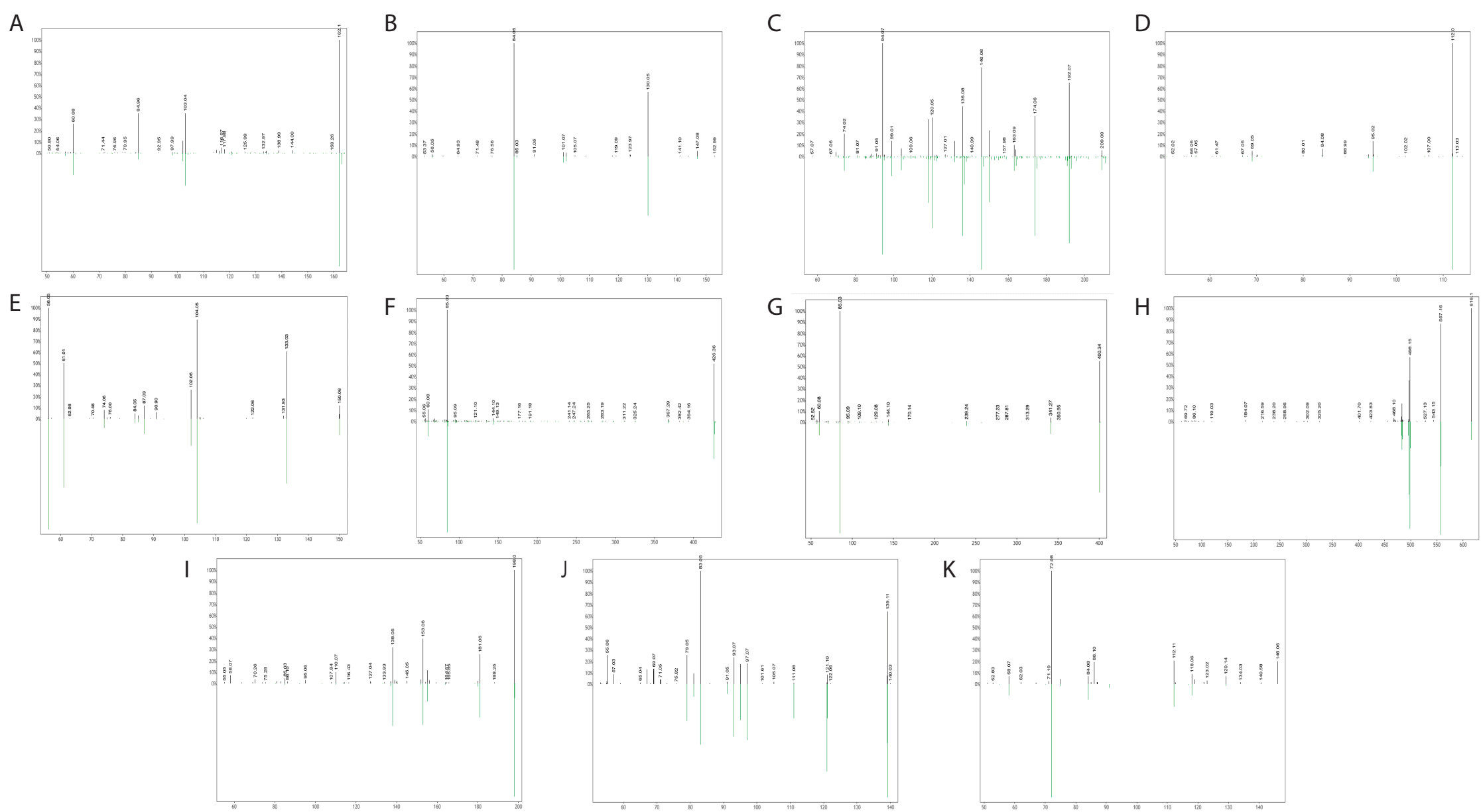

Supplement: FIG S2 [file msystems.00353-22-s0002.pdf]

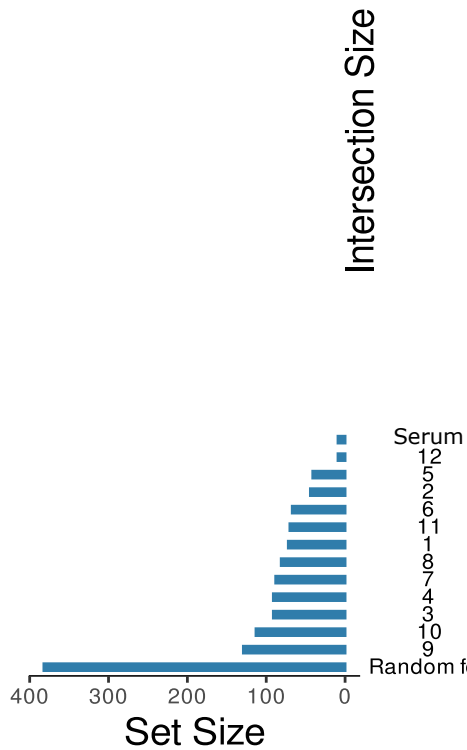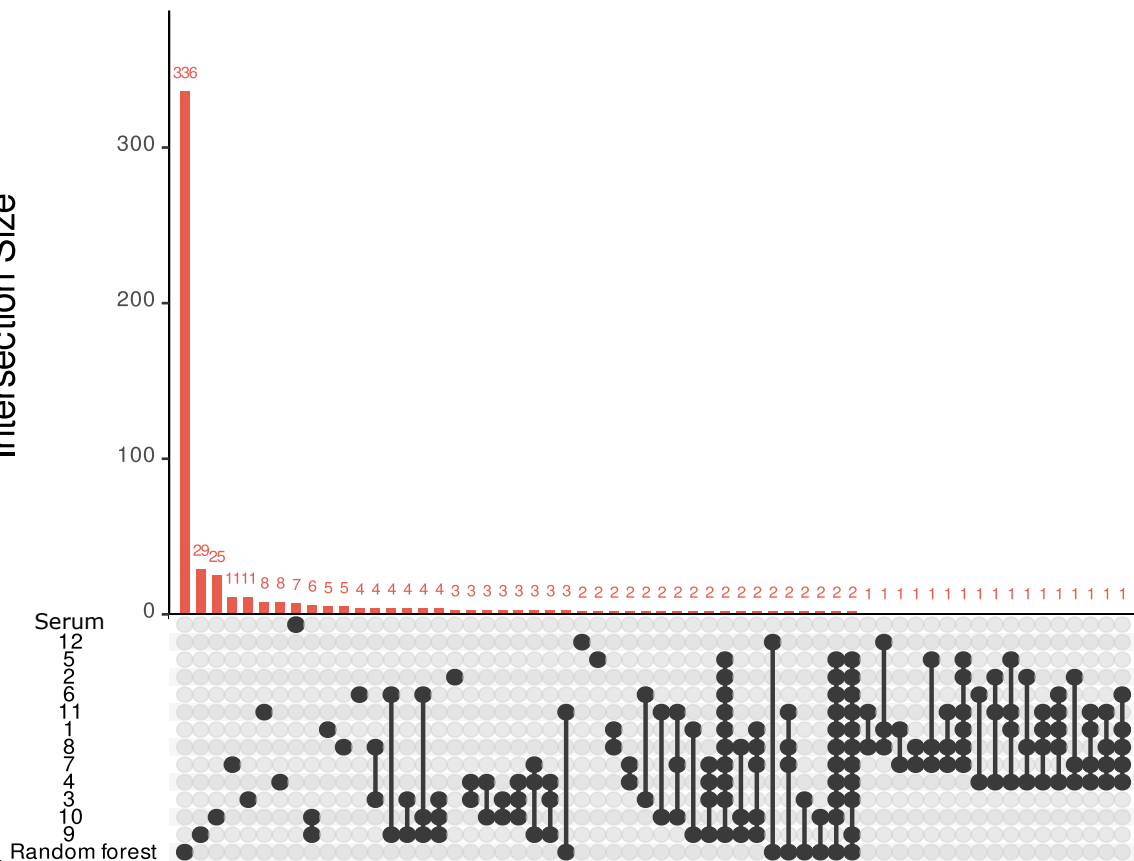

Supplement: FIG S3 [file msystems.00353-22-s0003.pdf]

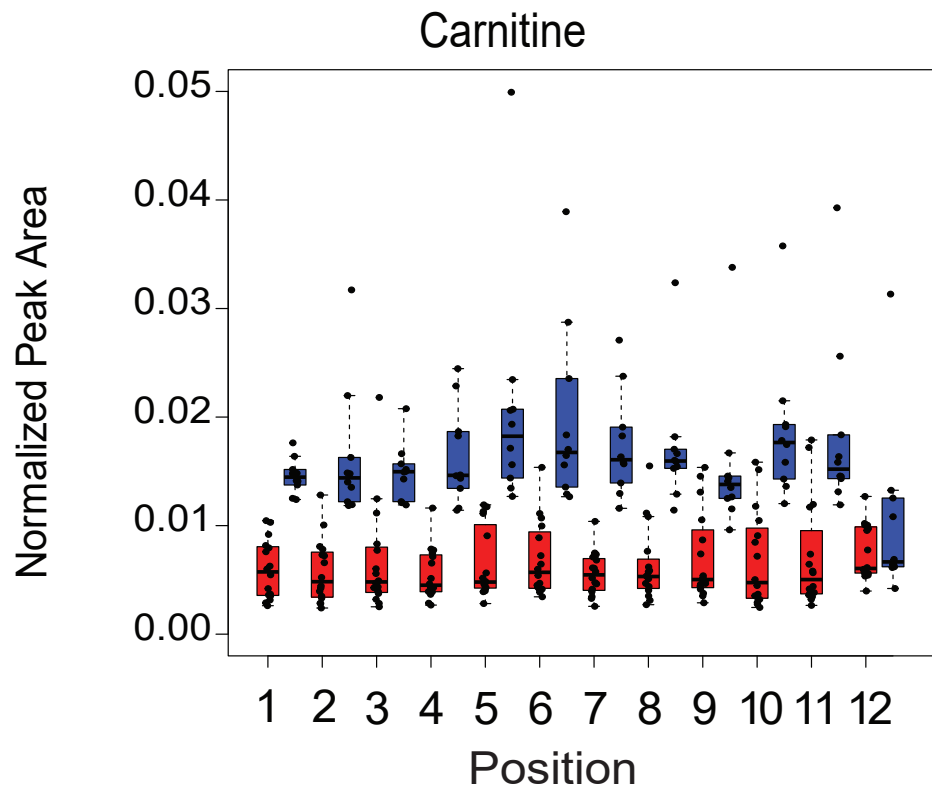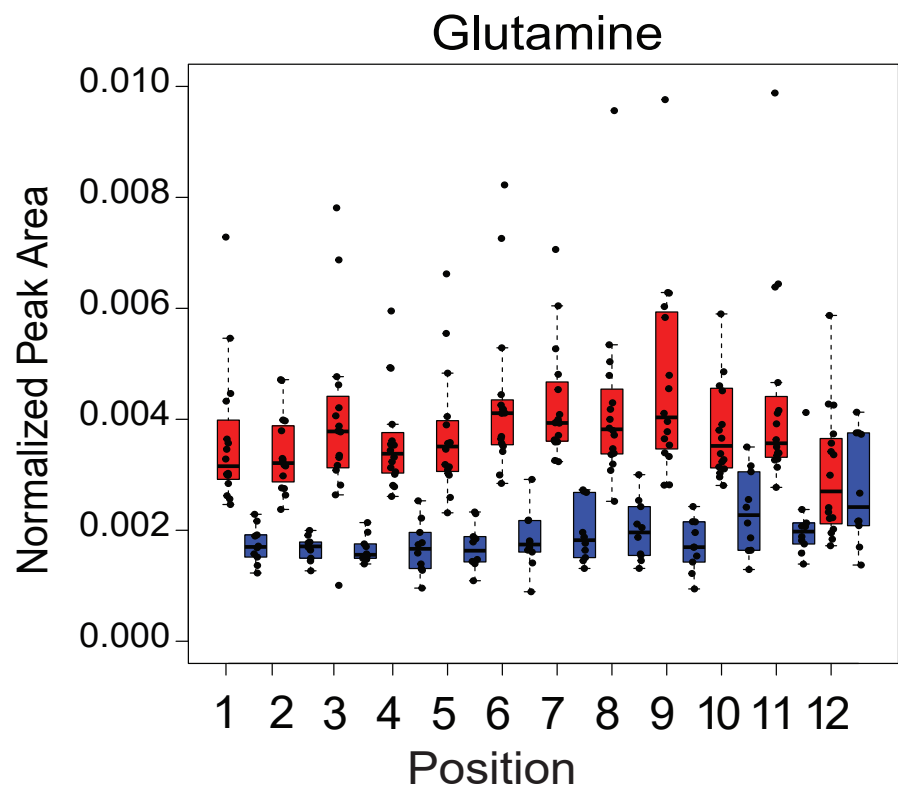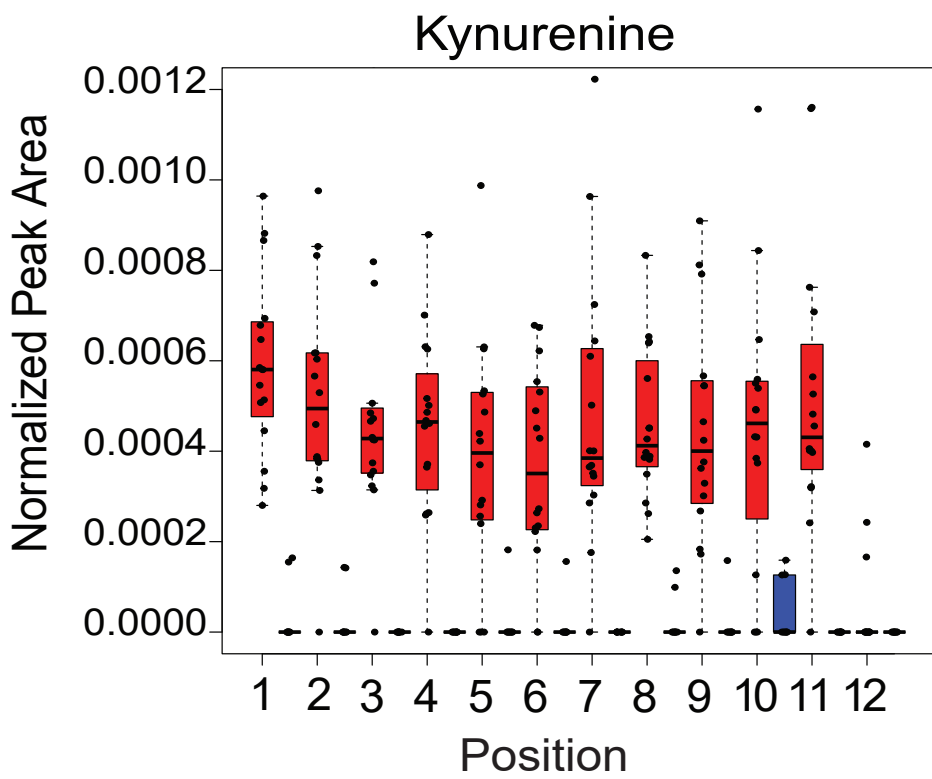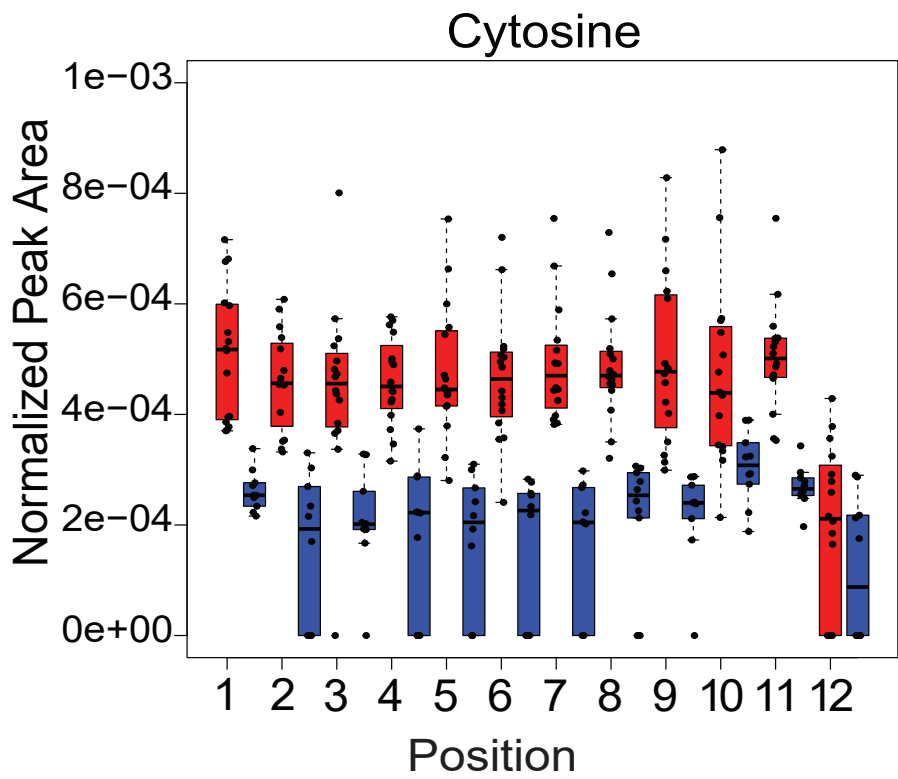

Supplement: FIG S1 [file msystems.00353-22-s0001.pdf]

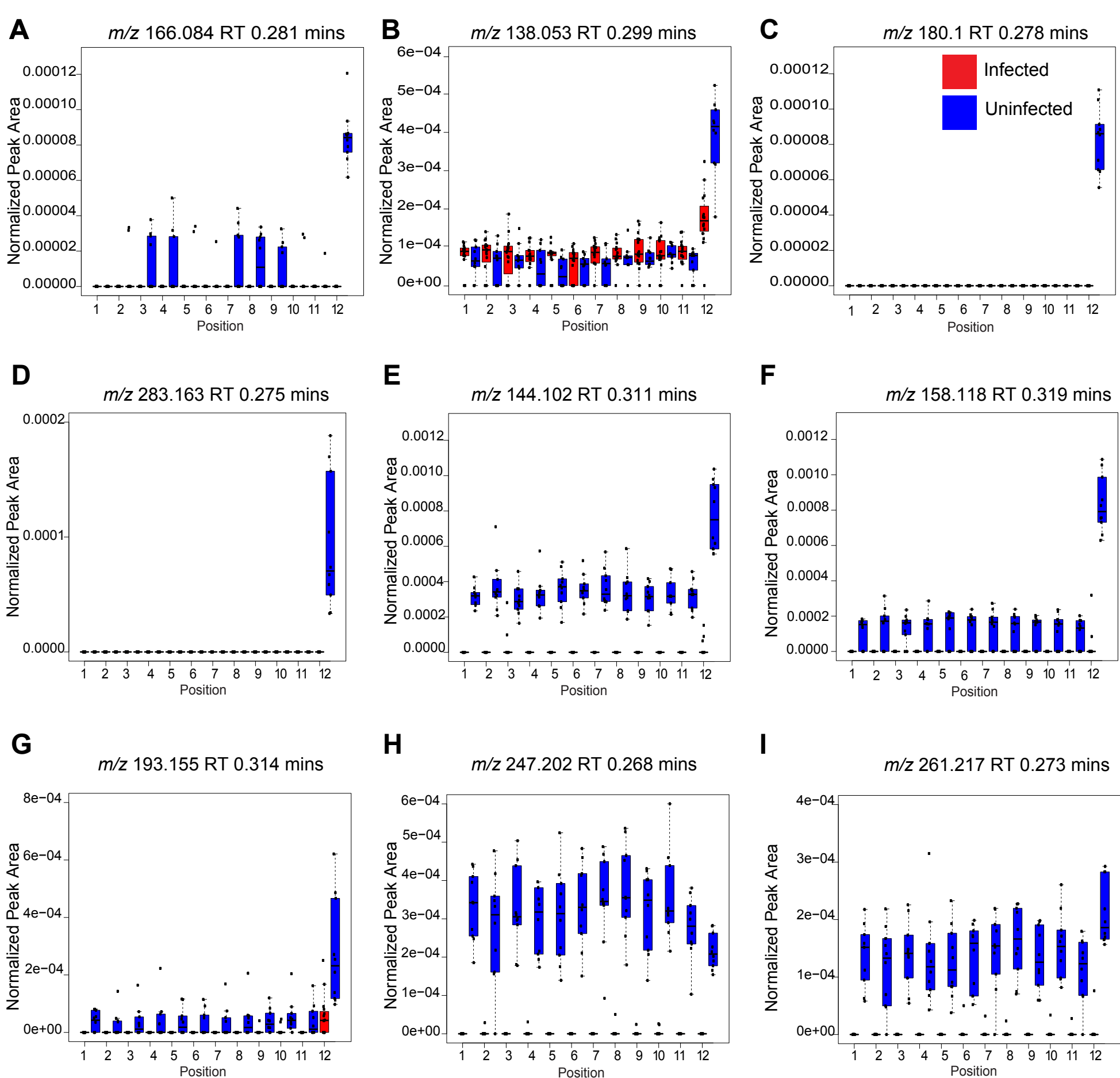

Supplement: FIG S4 [file msystems.00353-22-s0004.pdf]

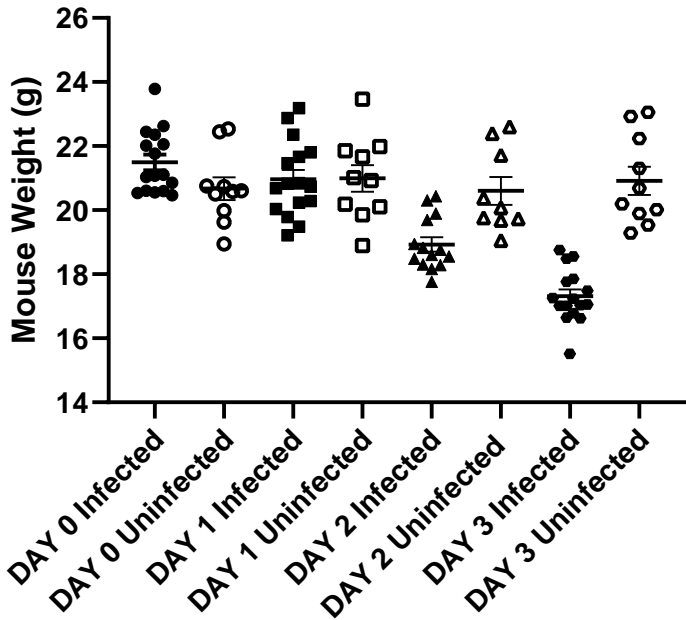

Supplement: FIG S5 [file msystems.00353-22-s0005.pdf]

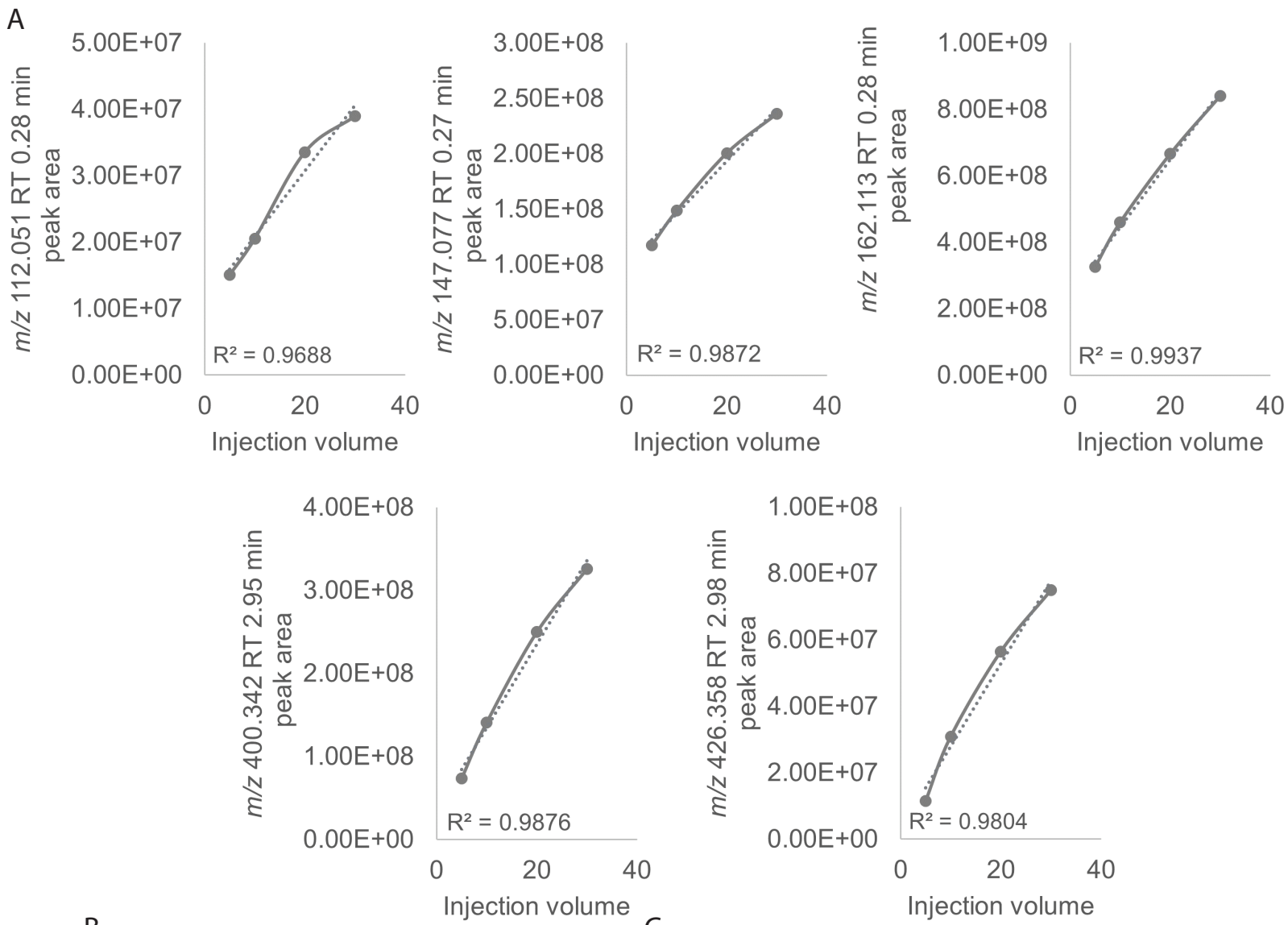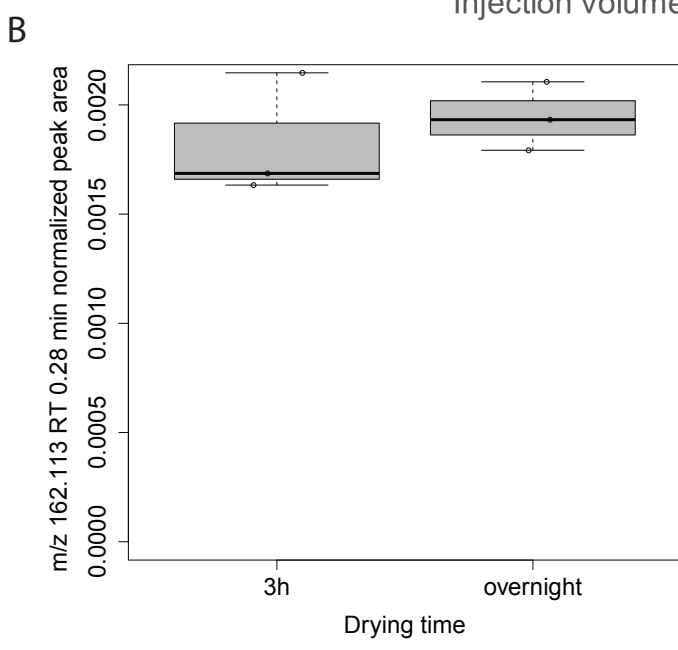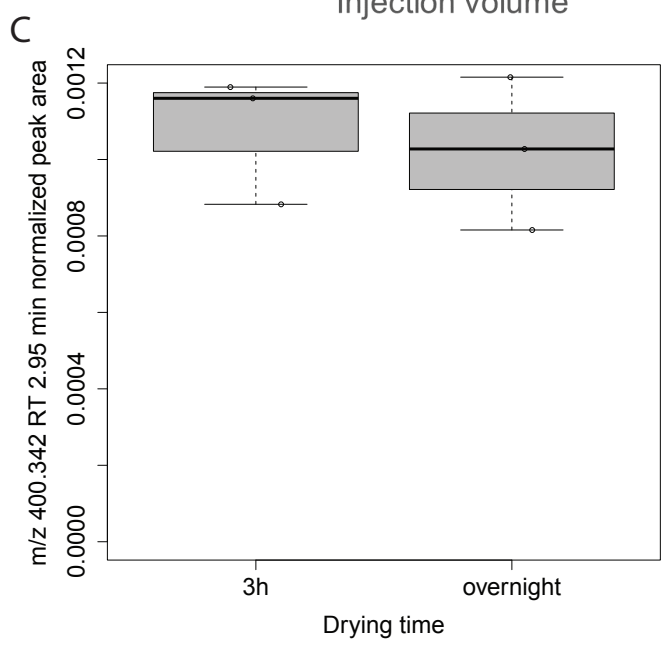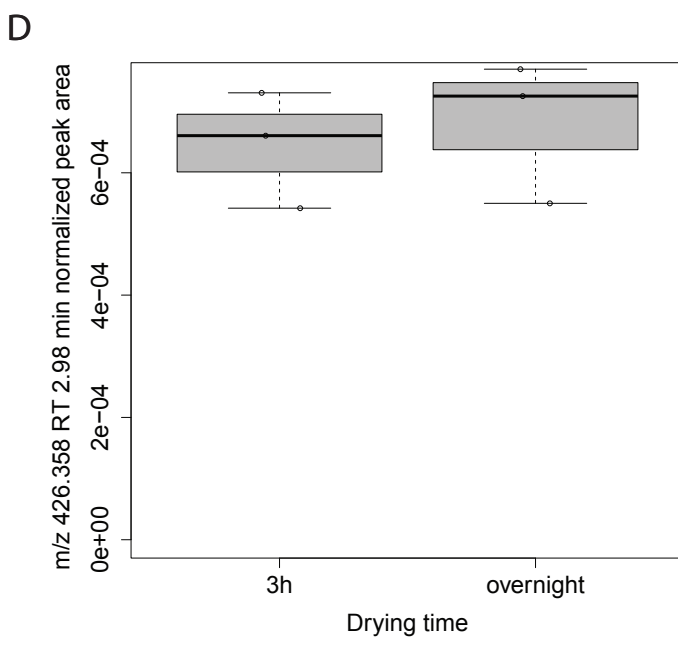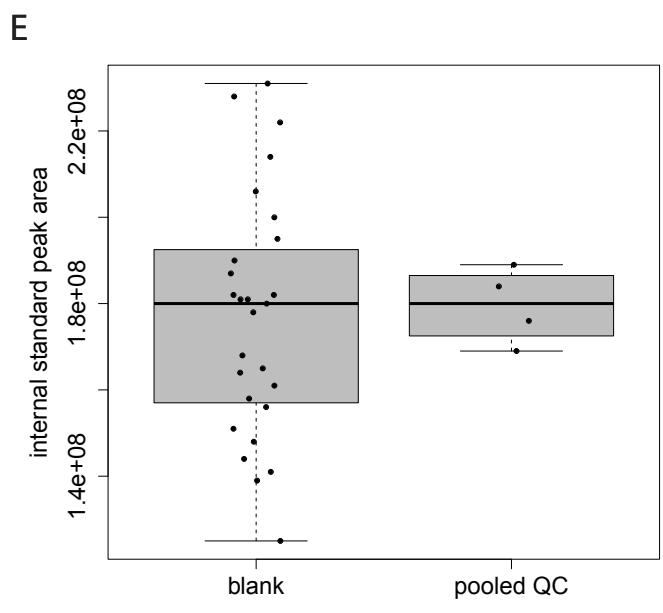

Supplement: FIG S6 [file msystems.00353-22-s0006.pdf]

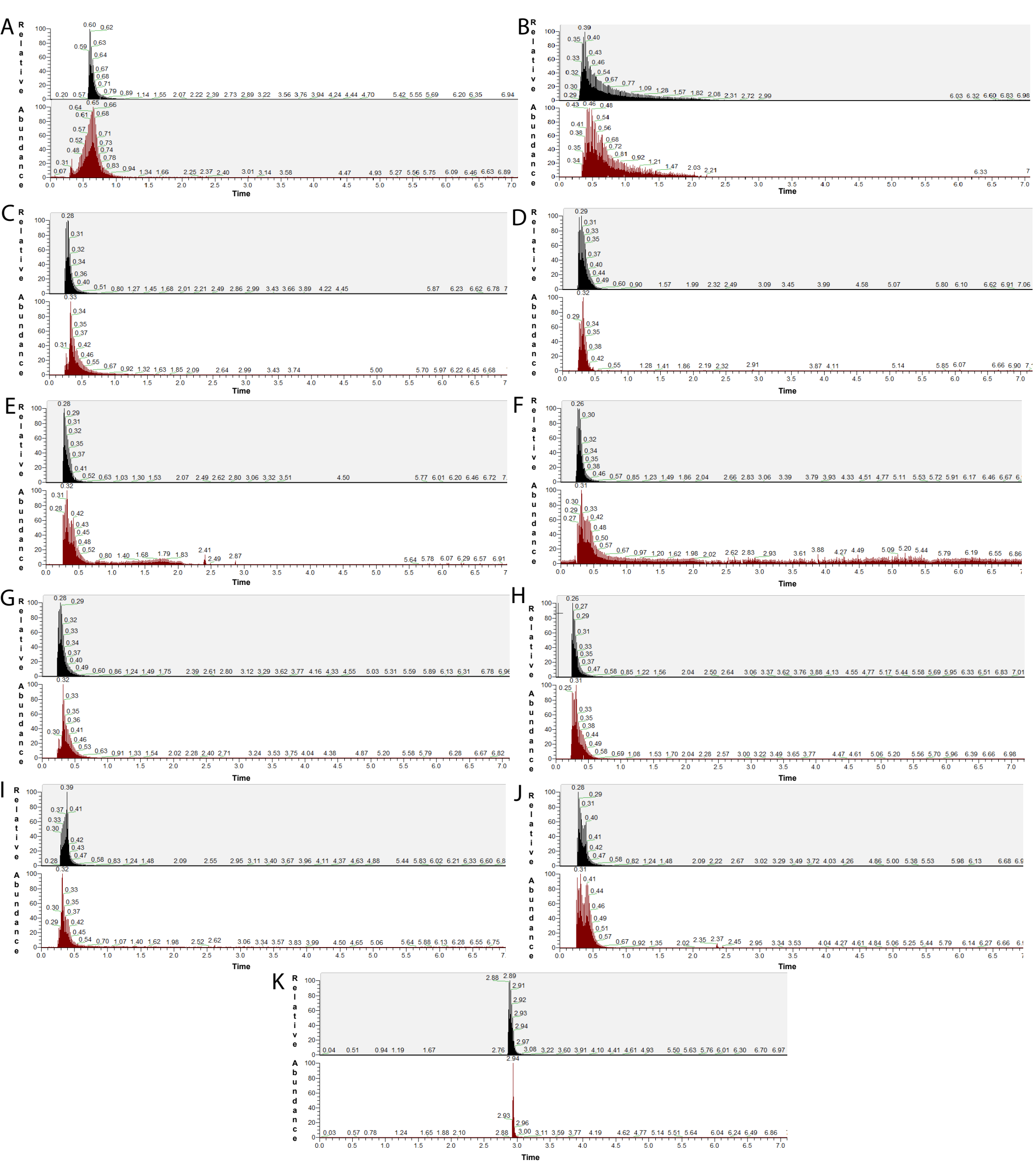

Supplement: FIG S7 [file msystems.00353-22-s0007.pdf]
